# Relationship of Blood Inflammatory Composite Markers with Cardiovascular Risk Factors and Subclinical Atherosclerosis in Patients with Rheumatoid Arthritis

**DOI:** 10.3390/life13071469

**Published:** 2023-06-28

**Authors:** Marta González-Sierra, Adrián Quevedo-Rodríguez, Alejandro Romo-Cordero, Gaël González-Chretien, Juan Carlos Quevedo-Abeledo, Antonia de Vera-González, Alejandra González-Delgado, Candelaria Martín-González, Miguel Ángel González-Gay, Iván Ferraz-Amaro

**Affiliations:** 1 Divsion of Hospitalization-at-Home, Hospital Universitario de Canarias, 38320 Tenerife, Spain; martagses@gmail.com; 2 Division of Rheumatology, Hospital Universitario Dr. Negrín, 35010 Las Palmas de Gran Canaria, Spain; adrian-ce@hotmail.es (A.Q.-R.); quevedojcarlos@yahoo.es (J.C.Q.-A.); 3 Division of Internal Medicine, Hospital Universitario de Canarias, 38320 Tenerife, Spain; alexromo96co@gmail.com (A.R.-C.); mmartgon@ull.edu.es (C.M.-G.); 4 Internal Medicine Department, Universidad de La Laguna, 38200 Tenerife, Spain; alu0101035901@ull.edu.es; 5 Division of Central Laboratory, Hospital Universitario de Canarias, 38320 Tenerife, Spain; adeverag@gmail.com (A.d.V.-G.); alejandra.gd88@gmail.com (A.G.-D.); 6 Division of Rheumatology, IIS-Fundación Jiménez Díaz, 28040 Madrid, Spain; 7 Department of Medicine and Psychiatry, Universidad de Cantabria, 39005 Santander, Spain; 8 Division of Rheumatology, Hospital Universitario de Canarias, 38320 Tenerife, Spain

**Keywords:** rheumatoid arthritis, blood composite inflammation scores, cardiovascular disease

## Abstract

The neutrophil-to-lymphocyte ratio (NLR), monocyte-to-lymphocyte ratio (MLR), platelet-to-lymphocyte ratio (PLR), and systemic immune-inflammatory index (SIRI, neutrophils × monocytes/lymphocytes) have been described as potential blood-derived inflammatory biomarkers in several diseases. Rheumatoid arthritis is an inflammatory disease that has been related to an increased risk of cardiovascular (CV) disease. In the present work, we analyze how these hematological composite scores of inflammation are related to classic CV risk factors and subclinical atherosclerosis in patients with RA. In this cross-sectional study that included 430 patients with RA, the NLR, MLR, PLR, and SIRI scores were calculated. Multivariable analysis was performed to examine the relationships of these composite blood scores with subclinical carotid atherosclerosis and with traditional cardiovascular factors, producing a complete profile of lipid molecules and insulin resistance or indices of beta-cell function, and a Systematic Coronary Risk Assessment (SCORE2) calculation. C-reactive protein and disease activity were significantly and positively associated with the four blood composite scores. SCORE2 was significantly associated with higher values of SIRI, NLR, and MLR, but not PLR. These relationships were maintained when SCORE 2 was considered categorical; patients in the very high CV risk category had higher values in all hematological composite scores, except PLR. In the multivariable analysis, SIRI and NLR were independently associated with higher levels of beta cell dysfunction. In conclusion, SCORE2 and the values of the hematological composite scores were positively correlated in patients with RA. In addition, there were some relationships of these scores with traditional CV risk factors, with their association with beta cell dysfunction being the most consistent.

## 1. Introduction

White blood cells are the largest group of inflammatory cells, and their various subtypes, including neutrophils, lymphocytes, and monocytes, are essential inflammatory markers. The role of platelets as a marker of inflammation has also been recognized, since they have different functions in inflammatory responses. In this regard, white blood cells and platelets are present in the chronic inflammatory environment, with a role in the secretion of cytokines, proteases, angiogenic factors, and chemokines [1]. For this reason, several blood composite scores have been proposed. The neutrophil/lymphocyte ratio (NLR), in addition to the platelet/lymphocyte ratio (PLR) and the lymphocyte/monocyte ratio (LMR), have been defined as simple biomarkers of systemic inflammation [2]. In addition, a modern inflammation-related biomarker named the systemic inflammation response index (SIRI) was released in 2016 and is calculated as neutrophils × monocytes/lymphocytes [3]. All of these have been described to be highly sensitive measures of inflammation in various inflammatory diseases and conditions, such as cancer, cardiovascular disease, or infection [4,5,6].

Rheumatoid arthritis (RA) is an inflammatory, peripheral polyarthritis of unknown origin that predominantly affects multiple joints in a symmetric pattern. RA is considered an inflammatory state in which acute-phase reactants are elevated. Furthermore, the magnitude of the increase in these acute-phase reactants is directly associated with the extent of inflammation and synovitis. Certainly, the increased risk of cardiovascular (CV) morbidity and mortality in RA has been recognized, with accumulating evidence that RA is an independent CV risk factor [7]. Extensive studies have shown that inflammation plays an essential role in the pathogenesis of CV disease in patients with RA [8]. In this sense, in RA, leukocytes, platelets, and endothelial cells have the capability to release extracellular vesicles such as exosomes and microparticles. These vesicles can transport micro-RNA, auto-antigens, damage-associated molecular patterns, pro-inflammatory cytokines, and matrix metalloproteases. This transport mechanism may potentially contribute to heightened inflammation and vulnerability of atherosclerotic plaques in individuals with systemic inflammatory diseases [9].

In the present work we set out to analyze, in a large series of patients with RA, how the combined blood indices are related to traditional CV risk factors, including a complete lipid profile and indices of insulin resistance and subclinical atherosclerosis.

## 2. Materials and Methods

### 2.1. Study Participants

This was a cross-sectional study involving 430 consecutive patients diagnosed with rheumatoid arthritis (RA). All participants were aged 18 years or older and met the 2010 classification criteria established by the American College of Rheumatology (ACR) and the European League Against Rheumatism (EULAR) [9]. Rheumatologists diagnosed the patients, who received regular follow-up care at rheumatology outpatient clinics. To be included in this study, participants were required to have a minimum duration of RA disease of at least 1 year. As glucocorticoids are commonly used in RA treatment, patients taking prednisone at a dose equivalent to or less than 10 mg/day were eligible to participate. Several exclusion criteria were applied to this study. Patients with a history of myocardial infarction, angina, or stroke; a glomerular filtration rate below 60 mL/min/1.73 m^2^; cancer; or any other chronic diseases such as hypothyroidism, heart or respiratory diseases, and nephrotic syndrome were excluded. Additionally, patients with evidence of active infection were also excluded. The study protocol received approval from the Institutional Review Committees at the Hospital Universitario de Canarias and the Hospital Universitario Doctor Negrín, both located in Spain. All participants provided informed written consent (approval no. 2019-452-1). The research adhered to relevant guidelines and regulations, and the principles outlined in the Declaration of Helsinki.

### 2.2. Data Collection and Laboratory Assessments

Individuals included in the study completed a questionnaire regarding cardiovascular risk factors and medication use and underwent a physical examination. Body mass index (BMI), abdominal circumference, and systolic and diastolic blood pressure were assessed under standardized conditions. Information on smoking status, diabetes, and hypertension was collected. In patients diagnosed with rheumatoid arthritis (RA), disease activity was assessed through various measures, including the Disease Activity Score (DAS28) in 28 joints [10], the Clinical Disease Activity Index (CDAI) [11], and the Simple Disease Activity Index (SDAI) [12]. Additionally, disease-related disability was evaluated using the Health Assessment Questionnaire (HAQ) score [13]. To assess lipid profiles, enzymatic colorimetric assays were employed to determine levels of cholesterol, triglycerides, and HDL cholesterol. LDL cholesterol levels were calculated using the Friedewald formula. Furthermore, the erythrocyte sedimentation rate (ESR) and high-sensitivity C-reactive protein (CRP) were measured using standard techniques. The homeostatic model assessment (HOMA) method was employed to assess insulin resistance (IR), providing estimates of insulin sensitivity (%S) and β-cell function (%B) based on fasting plasma insulin, C peptide, and glucose concentrations. In this study, HOMA2, the updated computer HOMA model [14], was utilized. Hematological composite scores were calculated as follows: neutrophil-to-lymphocyte ratio (NLR) = neutrophils/lymphocytes; monocyte-to-lymphocyte ratio (MLR) = monocytes/lymphocytes; platelet-to-lymphocyte ratio (PLR) = platelets/lymphocytes; systemic inflammation response index (SIRI) = neutrophils × monocytes/lymphocytes. The units for neutrophils, monocytes, and lymphocytes were all 1000 cells/μL, and for platelets, the unit was 100,000 cells/μL. Blood samples were collected after fasting.

Cardiovascular risk score (SCORE2) was calculated according to the 2021 European Society of Cardiology guidelines on cardiovascular disease prevention in clinical practice [15]. SCORE2 categorizes risk as low-to-moderate, high, or very high based on different age groups (<50, 50–69, and ≥70 years). The SCORE2 scoring system is designed to estimate the 10-year risk of both fatal and non-fatal cardiovascular events in individuals between the ages of 40 and 69 years. However, for healthy individuals who are 70 years or older, the SCORE2-OP (older persons) algorithm provides estimates for both 5-year and 10-year risk of fatal and non-fatal cardiovascular events.

### 2.3. Carotid Ultrasound Assessment

Carotid ultrasound examination was conducted to assess carotid intima-media thickness (cIMT) in the common carotid artery and detect focal plaques in the extracranial carotid tree among patients with rheumatoid arthritis (RA) [16]. The Esaote Mylab 70 (Genoa, Italy), a commercially available scanner equipped with a 7–12 MHz linear transducer, and a real-time quality intima-media thickness (QIMT) automated software-guided radiofrequency technique (Esaote, Maastricht, The Netherlands), were utilized for the measurements. The assessment followed the criteria outlined in the Mannheim consensus [16] for plaque identification in the accessible extracranial carotid tree, including the common carotid artery, bulb, and internal carotid artery. Plaque criteria were defined as follows: a focal protrusion in the lumen with a cIMT measurement of at least >1.5 mm, a protrusion at least 50% larger than the surrounding cIMT, or an arterial lumen encroachment of >0.5 mm [17].

### 2.4. Statistical Analysis

Demographic and clinical characteristics of patients with RA are presented as means (standard deviation) or percentages for categorical variables. For continuous variables that did not follow a normal distribution, data are reported as medians and interquartile ranges (IQR). The association between disease-related data and blood composite scores (continuous dependent variable) was examined using multivariable linear regression analysis, with adjustments made for confounding variables. Multivariable beta coefficients in linear regression must be interpreted as the average amount by which the dependent variable increases when the independent variable increases by one and other independent variables are held constant. Confounders were selected from demographics and traditional CV risk factors if their *p*-values were below 0.20 in the univariable analysis of hematological scores. All analyses were conducted using Stata software, version 17/SE (StataCorp, College Station, TX, USA), with the two-sided significance level set at 5%. A *p*-value of less than 0.05 was considered statistically significant.

## 3. Results

### 3.1. Demographic and Disease-Related Data

A total of 430 patients with rheumatoid arthritis (RA) were included in this study. Table 1 presents the demographic and disease-related characteristics of the participants. The mean age was 55 ± 10 years, and 81% of the patients were female. The patients had a mean BMI of 29 ± 15 kg/m^2^ and an abdominal circumference of 97 ± 13 cm. Traditional cardiovascular (CV) risk factors were common among the patients, with 22% being current smokers, 13% having type 2 diabetes, 32% classified as obese (BMI ≥ 30 kg/m^2^), and 34% having hypertension. Additionally, 32% of the patients were taking statins. The mean carotid intima-media thickness (cIMT) was 696 ± 131 microns, and 42% of the patients had carotid plaques. The median SCORE2 value for the entire population was 3.7 (IQR 1.8–5.9)%. According to SCORE2, 62% of the subjects were categorized as having low or moderate CV risk, while 25% and 13% were classified as having high and very high CV risk, respectively. Table 1 also provides information on the SIRI, NLR, MLR, and PLR values, full lipid profile molecules, and insulin resistance indices.

In the studied cohort, the median duration of the disease was 8 years, with an interquartile range (IQR) of 4 to 15 years. At the time of the study, the mean level of C-reactive protein (CRP) was 2.7 mg/L, with an IQR of 1.3 to 6.1 mg/L, and the mean erythrocyte sedimentation rate (ESR) was 18 mm/1st h, with an IQR of 7 to 32 mm/1st h. A total of 72% of the patients tested positive for rheumatoid factor, while 65% tested positive for anti-citrullinated protein antibodies. Disease activity, as assessed by the Disease Activity Score based on 28 joints and ESR (DAS28-ESR), had a mean value of 3.1 ± 1.4. Prednisone was being used by 36% of the patients, and 87% were taking at least one conventional disease-modifying antirheumatic drug (DMARD), with methotrexate being the most frequently prescribed DMARD (73%). Additionally, 19% of the patients were undergoing anti-tumor necrosis factor therapy. For further details on treatment frequencies and historical disease-related data, please refer to Table 1.

The median duration of the disease in our cohort of was 8 (IQR 4–15) years. The mean values of CRP and ESR, at the time the study was performed, were 2.7 (IQR 1.3–6.1) mg/L and 18 (IQR 7–32) mm/1st h, respectively. Seventy-two percent of patients were positive for rheumatoid factor, and 65% for anti-citrullinated protein antibodies. Disease activity measured via DAS28-ESR was 3.1 ± 1.4. Thirty-six percent of the patients were on prednisone and 87% were taking at least one conventional disease-modifying antirheumatic drug (DMARD), with methotrexate being the most widely used (73%). Nineteen percent of the patients were receiving anti-tumor necrosis factor therapies. The frequency of use of other treatments and historical disease-related data are shown in Table 1.

### 3.2. Association of Cardiovascular Risk Factors, SCORE2, and Disease-Related Data with Blood Composite Scores

**Figure 1:**
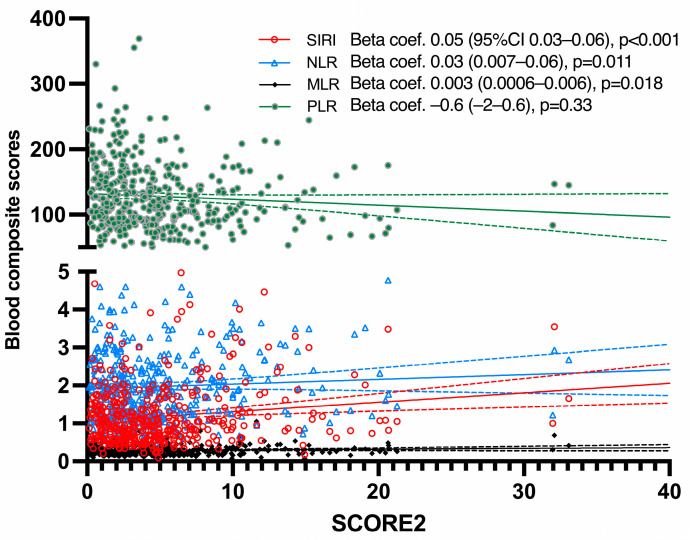
Relationship of SCORE2 with blood composite scores. neutrophil-to-lymphocyte ratio (NLR), monocyte-to-lymphocyte ratio (MLR), platelet-to-lymphocyte ratio (PLR) and systemic immune-inflammatory index (SIRI, neutrophils × monocytes/lymphocytes).

The relationship of CV risk factors, including SCORE2 and disease-related data, to hematological markers (as dependent variables) is shown in Table 2. Female patients revealed significantly lower values of SIRI, NLR, and MLR compared to males. CV risk factors, such as smoking, hypertension, the presence of type 2 diabetes, and BMI or abdominal adiposity, were not generally associated with blood inflammation composite scores. Only smoking and diabetes were significantly associated with lower PLR and higher SIRI levels, respectively. Despite this, SCORE2 was highly and significantly related to higher values of SIRI, NLR, and MLR, but not PLR (Figure 1). These relationships held when SCORE2 was considered categorical. In this sense, patients in the very high CV risk category revealed higher values in all hematological composite scores, except PLR (Table 2).

About disease-related data, several significant associations were found between disease characteristics and hematological inflammation markers (Table 2). In this sense, CRP was significantly and positively associated with all blood composite scores. This was also the case for ESR, except for PLR. Regarding disease activity scores, SDAI showed a positive and significant association with all four scores. On the contrary, CDAI did not show a relationship with any of them, and DAS28-ESR and DAS28-CRP only showed a significant relationship with NLR. Regarding disease treatments, the use of prednisone was associated with higher values of SIRI and NLR. In addition, significant negative relationships for NLR were found in patients receiving leflunomide, anti-TNF therapy and tocilizumab; and for MLR in those on therapy with hydroxychloroquine, salazopyrin, and anti-TNF (Table 2).

### 3.3. Multivariable Analysis of the Relationship of Blood Composite Scores with the Lipid Profile and the Indices of Insulin Resistance

The influence of peripheral blood scores (as independent variable) on lipid pattern molecules and insulin resistance indices is shown in Table 3. Regarding lipid profile, although some relationships were observed in univariate analysis, only PLR was significantly associated with higher serum apolipoprotein C-III levels after adjustment for covariates.

The relationship of hematological composite scores with glucose homeostasis molecules and insulin resistance indices was only evaluated in non-diabetic patients and if glucose was less than 110 mg/dL (n = 338). In this subgroup, after multivariable analysis, SIRI and NLR were associated with significant and higher levels of beta cell function—HOMA2-B%. Furthermore, no associations were found between carotid IMT and plaque and blood inflammation scores (Table 3).

## 4. Discussion

In the present work, we studied, for the first time, the relationships of several composite blood scores with the CV comorbidity that occurs in patients with RA. According to our results, these markers correlate with SCORE2, and higher values of them are associated with beta cell dysfunction in this population. We therefore believe that hematology score levels, which reflect inflammatory status, may be useful diagnostic surrogate biomarkers for CV disease in patients with RA.

RA patients have been reported to have higher peripheral blood score values than controls. In a recent meta-analysis comprising thirteen studies on neutrophil-to-lymphocyte ratio (NLR) with a total of 1550 patients with RA and 1128 healthy controls, as well as eight studies on platelet-to-lymphocyte ratio (PLR) with 380 RA patients and 305 healthy controls, both NLR and PLR were found to be significantly elevated in RA patients compared to the control groups. This was also the case in a study of 1499 RA patients from five hospitals and 366 healthy volunteers serving as controls, in which NLR, MLR, PLR, and SIRI were significantly increased in RA patients [10]. Furthermore, in a study using data from the NHANES database from 1999 to 2018, which included a total of 37,604 patients, of whom 2642 (7%) had rheumatoid arthritis, after adjusting for all covariates, multivariable logistic regression analysis showed that high SIRI levels were associated with a higher probability of RA [11].

However, there is some controversy regarding the relationship between hematologic composite scores and disease activity in RA. In a study involving 547 adults diagnosed with RA, it was observed that patients with active disease displayed higher neutrophil-to-lymphocyte ratios (NLRs) and platelet-to-lymphocyte ratios (PLRs) compared to those in remission. However, the discriminatory ability of NLR and PLR in detecting disease activity was found to be lower than that of CRP and erythrocyte sedimentation rate ESR. Additionally, the combination of NLR and PLR did not significantly enhance the diagnostic value of CRP and ESR [12]. Based on this study, it appears that NLR, PLR, and MLR are not useful independent or complementary diagnostic markers for disease activity in patients with RA. However, in a meta-analysis that included 18 studies, NLR and PLR were able to significantly discriminate RA patients with and without active disease [13]. In addition, some peripheral blood scores have been shown to decrease after RA treatment [14] or to be able to predict failure of triple therapy [15]. Our study coincides with these previously mentioned data. In this sense, in our cohort, the four scores were significantly related to CRP, and three of them, except PLR, to the ESR. In addition, all of them also showed a relationship with SDAI, a disease activity score that contains information on swollen and painful joints and includes CRP. This relationship was less consistent with other disease activity scores. In addition, the use of various disease treatments was associated with lower hematology scores.

SCORE2, a state-of-the-art algorithm incorporating conventional cardiovascular risk factors, is widely utilized to estimate the 10-year risk of developing cardiovascular disease for the first time. In our work, SCORE2 showed a positive and significant association with higher values of SIRI, NLR, and MLR, but not PRL. This relationship was also significant when SCORE2 was considered categorical and hematology score levels were compared between the very high and low or moderate risk categories. In this regard, we are not aware of any study that has studied this relationship in the general population. However, the predictive ability of peripheral blood scores for CV events has recently been reviewed. After analyzing a total of 13 studies with 152,996 participants, the overall pooled results showed that SIRI was significantly associated with an increased risk of CV disease [16]. This increased risk could be observed in almost all CV disease subtypes, including ischemic stroke, hemorrhagic stroke, myocardial infarction, and peripheral arterial disease. Similar but non-significant trends were found for venous thrombosis, small-blood-vessel disease, and acute coronary syndrome. Furthermore, the pooled results showed that SIRI levels at CV disease onset were significantly higher than those in the general population, which was consistent across different CV disease subtypes. However, the quality of the evidence was generally considered low in this meta-analysis. The authors suggested further well-designed studies to determine the optimal cut-off value and characterize the population that would benefit [16]. Similar evidence has been reported in RA.

In relation to this, a retrospective study involving 48 patients with RA found that SIRI was independently linked to the development of ischemic stroke in individuals with RA [16]. The fact that, in our study, SIRI, NLR, and MLR were related to SCORE2 in RA patients suggests that these markers may be used to assess CV risk in these patients, and that CV risk factor prevention should be kept in mind in those RA patients with higher values of these markers.

In our study, we found no relationship between hematological scores and lipid profiles, but we did find a relationship between SIRI and NLR and greater beta cell dysfunction after multivariable adjustment. It is known that RA patients have insulin resistance and that this is partly explained by the inflammatory state of the disease [17]. Additionally, blood cell count has been linked to insulin resistance and diabetes in the general population in previous reports. In this regard, in a recent study involving a sample of 1470 patients with normoglycemia, 1124 with pre-diabetes, and 396 with type 2 diabetes, white blood cell count was found to be associated with both HOMA-IR and HOMA-B%, with a stronger correlation observed in HOMA-B% after adjusting for covariates. Similarly, platelet count showed a correlation with HOMA-IR and HOMA-B%, but its significance persisted only in HOMA-B% after conducting multivariable analysis [18]. Similarly, a higher white blood cell count seems to predict the development of impaired fasting glucose or type 2 diabetes mellitus [19]. Our findings in RA were similar to those described in the healthy population. However, it is important to note that we performed this analysis in RA patients with normal fasting blood glucose. Therefore, the association with beta cell dysfunction described in our study may indicate that higher values of blood cell markers in RA patients may predict the existence of a prediabetic state.

Finally, we did not observe a relationship between hematological scores and the presence of carotid plaque or cIMT. Therefore, the role of CV risk factors in the development of atherosclerotic disease in RA is clearly greater than that of hematological markers.

Several studies have studied the relationship of blood composite scores with CV disease in the general population. In a report of 158 patients with chronic kidney disease, red blood cell distribution width correlated with CRP, central diastolic blood pressure, and albuminuria [20]. Additionally, NLR correlated with the duration of cardiovascular disease, CRP, central diastolic blood pressure, and glomerular filtration rate. The authors concluded that red blood cell distribution width and NLR could serve as predictors of traditional and novel markers of vascular calcification and CV disease [20]. Similarly, in a study that enrolled 74 patients, calcium kidney stones were associated with higher levels of inflammatory biomarkers, including NLR, MLR, and SIRI, compared with age- and sex-matched non-stone formers [21]. Also, in this report, after stratification by CV risk category, all inflammatory biomarkers and the severity of carotid artery stenosis were still significantly higher in calcium stone formers. Another study aimed to analyze the preoperative role of NLR and PLR in the medium-term outcome of patients surgically revascularized for femoropopliteal disease. The authors concluded that higher preoperative NLR and PLR values determined upon hospital admission were strongly predictive of 12-month primary patency failure, and of a higher amputation rate and fatality, for all patients enrolled in the study [22] Lastly, NLR levels have proven to help in the prediction of death in patients with hepatocellular carcinoma after trans-arterial chemoembolization [23]. Our study focused on the relationship between hematological scores and traditional CV risk factors or CV disease calculators. Nevertheless, our conclusions are in line with the studies previously discussed that support the prognostic and diagnostic value of these blood biomarkers.

There are several limitations to our study. One of them is the absence of a control group. However, many studies have already explored the role of blood composite scores in CV disease, the prediction of CV events, and other CV comorbidities in the general population. Second, we could not evaluate the causal relationship between hematological score parameters and the outcomes of our study due to its cross-sectional design. Third, the exact biologic mechanisms underlying the associations described in our work need to be clarified by further studies. Fourth, we think that the combination of some of these scores could be helpful in measuring systemic inflammation. However, there are still no studies that have validated these combinations. Fifth, although we registered the use of antihypertensive therapy, we did not consider the types of diuretics used by the subjects.

## 5. Conclusions

In conclusion, hematological composite scores are related to SCORE2 risk calculations and to several CV comorbidities in patients with RA.

## Figures and Tables

**Table 1:** Demographics, cardiovascular risk factors, and disease-related data of RA patients.

	Rheumatoid Arthritis (n = 430)
Age, years	55 ± 10
Female, n (%)	350 (81)
BMI, kg/m^2^	29 ± 15
Abdominal circumference, cm	97 ± 13
Blood cell composite score	
Systemic inflammation response index (SIRI)	1.22 ± 0.81
Neutrophil-to-lymphocyte ratio	2.01 ± 1.02
Monocyte-to-lymphocyte ratio	0.29 ± 0.12
Platelet-to-lymphocyte ratio	131 ± 55
Cardiovascular risk factors and data	
Current smoker	93 (22)
Obesity	137 (32)
Hypertension	148 (34)
Diabetes mellitus	54 (13)
Dyslipidemia	200 (47)
Statins, n (%)	139 (32)
SCORE2, %	3.7 (1.8–5.9)
Low or moderate risk	265 (62)
High risk	108 (25)
Very high risk	57 (13)
Laboratory	
Total cholesterol, mg/dL	205 ± 38
Triglycerides, mg/dL	147 ± 86
HDL cholesterol, mg/dL	57 ± 15
LDL cholesterol, mg/dL	120 ± 34
LDL:HDL cholesterol ratio	2.27 ± 0.93
Non-HDL cholesterol, mg/dL	149 ± 39
Lipoprotein (a), mg/dL	34 (11–107)
Apolipoprotein A1, mg/dL	173 ± 31
Apolipoprotein B, mg/dL	106 ± 26
ApoB:Apo A ratio	0.63 ± 0.24
Apolipoprotein C3, mg/dL	4.8 (2.2–8.7)
Glucose, mg/dL	95 ± 24
Insulin, µU/ml	8.6 (5.5–15.1)
C-peptide, ng/ml	2.5 (1.6–4.0)
HOMA2-IR	1.09 (0.7–2.0)
HOMA2-S%	92 (51–142)
HOMA2-B%-C-peptide	162 ± 77
Disease-related data	
Disease duration, years	8 (4–15)
CRP at time of study, mg/L	2.7 (1.3–6.1)
ESR at time of study, mm/1º h	18 (7–32)
IL-6, pg/ml	5.0 (3.2–8.6)
Rheumatoid factor, n (%)	303 (72)
ACPA, n (%)	253 (65)
DAS28-ESR	3.13 ± 1.35
DAS28-PCR	2.73 ± 1.08
SDAI	12 (7–19)
CDAI	8 (4–14)
History of extra-articular manifestations, n (%)	38 (10)
Erosions, n (%)	166 (43)
Current drugs, n (%)	
Prednisone	155 (36)
Prednisone doses, mg/day	5 (3–5)
NSAIDs	194 (45)
DMARDs	373 (87)
Methotrexate	316 (73)
Leflunomide	94 (22)
Hydroxychloroquine	45 (18)
Salazopyrin	28 (7)
Anti TNF therapy	83 (19)
Tocilizumab	23 (5)
Rituximab	7 (2)
Abatacept	12 (3)
JAK inhibitors	20 (5)
Carotid ultrasound	
cIMT, microns	696 ± 131
Carotid plaque, n (%)	180 (42)

Data represent means ± SD or medians (IQR) when data are not normally distributed. CV: cardiovascular; LDL: low-density lipoprotein; HDL: high-density lipoprotein; CRP: C-reactive protein; TNF: tumor necrosis factor; ESR: erythrocyte sedimentation rate; NSAID: non-steroidal anti-inflammatory drugs; DMARD: disease-modifying antirheumatic drug; SCORE: Systematic Coronary Risk Evaluation; cIMT: carotid intima-media thickness; BMI: body mass index; DAS28: Disease Activity Score in 28 joints; ACPA: anti-citrullinated protein antibodies; HOMA: homeostatic model assessment; CDAI: Clinical Disease Activity Index; SDAI: Simple Disease Activity Index.

**Table 2:** Demographics, cardiovascular risk factors, and disease-related data in relation to SIRI.

	Beta Coef. (95%CI), *p*							
	SIRI		NLR		MLR		PLR	
Age, years	0.004 (−0.004–0.01)	0.34	−0.003 (−0.01–0.007)	0.61	0.0002 (−0.001–0.001)	0.75	**−0.6 (−1–(−0.1))**	**0.019**
Female, n (%)	**−0.5 (−0.7–(−0.3))**	**<0.001**	**−0.4 (−0.7–(−0.2))**	**0.001**	**−0.05 (−0.08–(−0.02))**	**<0.001**	3 (−11–16)	0.69
BMI, kg/m^2^	−0.0009 (−0.006–0.004)	0.73	−0.004 (−0.01–0.003)	0.25	0.00007 (−0.0007–0.0008)	0.86	0.01 (−0.3–0.4)	0.95
Abdominal circumference, cm	**0.006 (0.0002–0.01)**	**0.043**	0.0006 (−0.007–0.008)	0.87	0.0004 (−0.0005–0.001)	0.35	−0.2 (−0.6–0.3)	0.47
Cardiovascular data								
CV risk factors, n (%)								
Current smoker	0.1 (−0.04–0.3)	0.14	−0.03 (−0.3–0.2)	0.82	−0.03 (−0.06–0.0008)	0.057	**−20 (−33–(−7))**	**0.002**
Obesity	−0.02 (−0.2–0.2)	0.85	−0.2 (−0.4–0.05)	0.14	−0.0004 (−0.03–0.03)	0.98	3 (−8–15)	0.58
Hypertension	0.1 (−0.06–0.3)	0.23	0.04 (−0.2–0.2)	0.72	0.01 (−0.01–0.04)	0.37	4 (−7–16)	0.45
Diabetes mellitus	**0.3 (0.09–0.6)**	**0.007**	0.3 (−0.008–0.6)	0.056	0.01 (−0.03–0.05)	0.59	2 (−14–18)	0.79
Dyslipidemia	−0.01 (−0.2–0.1)	0.86	−0.2 (−0.3–0.05)	0.13	−0.008 (−0.03–0.02)	0.50	−5 (−16–5)	0.33
Statins, n (%)	−0.03 (−0.2–0.1)	0.76	−0.1 (−0.3–0.09)	0.25	−0.009 (−0.03–0.02)	0.48	−2 (−14–9)	0.67
SCORE2, %	**0.05 (0.03–0.06)**	**<0.001**	**0.03 (0.007–0.06)**	**0.011**	**0.003 (0.0006–0.006)**	**0.018**	−0.6 (−2–0.6)	0.33
Low or moderate risk	ref.		ref.		ref.		ref.	
High risk	0.2 (−0.02–0.3)	0.082	0.2 (−0.06–0.4)	0.16	0.002 (−0.03–0.03)	0.89	**−14 (−26–(−1))**	**0.030**
Very high risk	**0.5 (0.3–0.8)**	**<0.001**	**0.3 (0.01–0.6)**	**0.041**	**0.05 (002–0.09)**	**0.004**	11 (−5–27)	0.17
High and very high risk	**0.1 (0.06–0.2)**	**<0.001**	**0.1 (0.008–0.2)**	**0.035**	0.01 (−0.002–0.02)	0.12	−3 (−8–3)	0.36
Disease-related data								
Disease duration, years	−0.008 (−0.02–0.001)	0.085	**−0.01 (−0.02–(−0.001))**	**0.027**	−0.001 (−0.003–0.00007)	0.063	−0.3 (−0.9–0.3)	0.27
CRP, mg/l	**0.01 (0.006–0.02)**	**<0.001**	**0.01 (0.004–0.02)**	**0.002**	**0.002 (0.0006–0.002)**	**0.001**	0.6 (−0.2–1)	0.006
ESR, mm/1º h	**0.007 (0.003–0.01)**	**<0.001**	**0.009 (0.004–0.01)**	**0.001**	**0.0008 (0.0002–0.001)**	**0.008**	0.2 (−0.05–0.5)	0.12
IL−6, pg/ml	−0.002 (−0.007–0.003)	0.44	−0.002 (−0.008–0.005)	0.62	−0.0002 (−0.001–0.0006)	0.61	−0.02 (−0.4–0.3)	0.91
Rheumatoid factor, n (%)	0.07 (−0.1–0.2)	0.44	0.2 (−0.06–0.4)	0.15	0.01 (−0.02–0.04)	0.44	8 (−4–20)	0.21
ACPA, n (%)	0.1 (−0.06–0.3)	0.20	0.1 (−0.1–0.3)	0.35	0.006 (−0.02–0.03)	0.57	5 (−7–17)	0.42
DAS28-ESR	0.05 (−0.007–0.1)	0.084	**0.1 (0.04–0.2)**	**0.002**	0.002 (−0.007–0.01)	0.71	3 (−0.9–7)	0.13
DAS28-PCR	0.04 (−0.03–0.1)	0.28	0.1 (0.02–0.2)	0.014	−0.0005 (−0.01–0.01)	0.93	3 (−2–8)	0.26
SDAI	**0.007 (0.003–0.01)**	**0.003**	**0.01 (0.004–0.02)**	**0.001**	**0.0009 (0.00009–0.002)**	**0.028**	**0.4 (0.05–0.7)**	**0.024**
CDAI	−0.002 (−0.01–0.008)	0.71	0.008 (−0.004–0.02)	0.20	−0.0007 (−0.002–0.0008)	0.35	0.07 (−0.6–0.8)	0.84
History of extra-articular manifestations, n (%)	0.07 (−0.2–0.3)	0.61	−0.07 (−0.4–0.3)	0.70	−0.0005 (−0.04–0.04)	0.98	4 (−15–23)	0.66
Erosions, n (%)	0.005 (−0.2–0.2)	0.96	−0.04 (−0.2–0.2)	0.71	0.003 (−0.02–0.03)	0.81	−1 (−12–10)	0.82
Current drugs, n (%)								
Prednisone	**0.2 (0.08–0.4)**	**0.003**	**0.3 (0.09–0.5)**	**0.004**	0.01 (−0.01–0.04)	0.29	0.2 (−11–11)	0.98
Prednisone doses, mg/day	−0.009 (−0.05–0.03)	0.66	−0.005 (−0.06–0.05)	0.84	−0.004 (−0–0.009–0.001)	0.14	−1 (−4–2)	0.38
NSAIDs	−0.1 (−0.3–0.002)	0.097	−0.1 (−0.3–0.09)	0.27	−0.02 (−0.04–0.003)	0.090	−3 (−13–8)	0.63
DMARDs	0.2 (−0.06–0.4)	0.15	0.1 (−0.2–0.4)	0.49	0.02 (−0.01–0.06)	0.19	4 (−11–20)	0.59
Methotrexate	0.1 (−0.07–0.3)	0.23	0.2 (−0.02–0.4)	0.068	−0.006 (−0.03–0.02)	0.68	2 (−10–14)	0.73
Leflunomide	−0.1 (−0.3–0.04)	0.13	**−0.4 (−0.6–(−0.1))**	**0.002**	0.01 (−0.02–0.04)	0.42	−2 (−15–10)	0.71
Hydroxychloroquine	−0.2 (−0.5–0.04)	0.088	−0.3 (−0.7–0.02)	0.063	**−0.05 (−0.09–(−0.008))**	**0.019**	−14 (−32–4)	0.14
Salazopyrin	−0.1 (−0.4–0.2)	0.53	−0.2 (−0.6–0.2)	0.31	**−0.05 (−0.1–(−0.002))**	**0.042**	−22 (−45–0.7)	0.057
Anti TNF therapy	−0.2 (−0.4–0.006)	0.057	**−0.3 (−0.6–(−0.07))**	**0.012**	**−0.04 (−0.7–(−0.008))**	**0.014**	**−18 (−32–(−5))**	**0.008**
Tocilizumab	−0.3 (−0.6–0.04)	0.084	**−0.4 (−0.9–(−0.005))**	**0.048**	−0.1 (−0.7–0.04)	0.58	−18 (−41–5)	0.13
Rituximab	0.5 (−0.1–1)	0.13	0.6 (−0.1–1)	0.098	**0.09 (−0.002–0.2)**	**0.046**	30 (−12–71)	0.16
Abatacept	0.1 (−0.4–0.6)	0.61	−0.1 (−0.8–0.5)	0.69	0.008 (−0.07–0.09)	0.83	−4 (−39–31)	0.81
JAK inhibitors	0.1 (−0.3–0.5)	0.59	0.07 (−0.4–0.5)	0.78	−0.002 (−0.06–0.05)	0.95	12 (−13–38)	0.35

Blood composite scores are considered the dependent variable in this analysis. SIRI: systemic inflammation response index; NLR: neutrophil-to-lymphocyte ratio; PLR: platelet-to-lymphocyte ratio; MLR: monocyte-to-lymphocyte ratio; CV: cardiovascular; CRP: C-reactive protein; ESR: erythrocyte sedimentation rate; NSAID: non-steroidal anti-inflammatory drugs; DMARD: disease-modifying antirheumatic drug; TNF: tumor necrosis factor; BMI: body mass index; DAS28: Disease Activity Score in 28 joints; ACPA: anti-citrullinated protein antibodies; cIMT: carotid intima-media thickness; CDAI: Clinical Disease Activity Index; SDAI: Simple Disease Activity Index. Significant *p*-values are depicted in bold.

**Table 3:** Hematological composite scores in relation to lipid profiles, insulin resistance indices, and carotid subclinical atherosclerosis.

	**SIRI**		**NLR**	
	**Univariable**	**Multivariable**	**Univariable**	**Multivariable**
	Beta coef. (95% CI)			
Lipid pattern				
Total cholesterol, mg/dL	−2 (−6–3), 0.44		−2 (−6–2), 0.26	
Triglycerides, mg/dL	0.5 (−10–11), 0.92		−5 (−14–3), 0.20	
HDL cholesterol, mg/dL	**−2 (−4–(−0.09)), 0.040**	−0.6 (−2–1), 0.54	−1 (−3–0.4), 0.14	−0.5 (−2–0.9), 0.48
LDL cholesterol, mg/dL	5 (−9–20), 0.46		4 (−7–16), 0.44	
LDL:HDL cholesterol ratio	0.2 (−0.04–0.4), 0.11	0.1 (−0.1–0.3), 0.28	0.1 (−0.04–0.3), 0.14	0.1 (−0.07–0.3), 0.26
Non-HDL cholesterol, mg/dL	0.1 (−5–5), 0.97		−1 (−5–3), 0.60	
Lipoprotein (a), mg/dL	6 (−4–15), 0.26		0.3 (−7–8), 0.95	
Apolipoprotein A1, mg/dL	**−4 (−8–(−0.3)), 0.036**	−3 (−7–1), 0.14	**−4 (−6–(−0.7)), 0.016**	−3 (−6–0.1), 0.060
Apolipoprotein B, mg/dL	0.6 (−5–6), 0.81		2 (−3–6), 0.47	
ApoB:Apo A ratio	0.02 (−0.009–0.05), 0.18	0.01 (−0.02–0.04), 0.39	0.02 (−0.003–0.04), 0.086	0.02 (−0.004–0.04), 0.11
Apolipoprotein C-III, mg/dL	**0.7 (0.03–1), 0.039**		0.4 (−0.06–0.9), 0.085	0.4 (−0.1–0.9), 0.12
Insulin resistance indices *				
Insulin, µU/mL	**1 (0.1–3), 0.035**	0.7 (−0.6–2), 0.27	0.6 (−0.3–2), 0.21	
C-peptide, ng/mL	**0.4 (0.05–0.7), 0.024**	0.2 (−0.2–0.5), 0.33	0.2 (−0.06–0.4), 0.14	0.1 (−0.09–0.3), 0.25
HOMA2-IR	**0.2 (0.0009–0.3), 0.049**	0.07 (−0.08–0.2), 0.35	0.06 (−0.05–0.2), 0.27	
HOMA2-S%	−6 (−17–6), 0.34		3 (−5–11), 0.47	
HOMA2-B%-C-peptide	**20 (8–30), 0.001**	**14 (3–25), 0.016**	**11 (3–19), 0.007**	**10 (2–18), 0.011**
Carotid ultrasound				
cIMT, microns	8 (−8–24), 0.31		0.2 (−12–13), 0.97	
Carotid plaque, n (%)	1.07 (0.84–1.36), 0.60		0.99 (0.81–1.19), 0.88	
	**MLR**		**PLR × 100**	
	**Univariable**	**Multivariable**	**Univariable**	**Multivariable**
Lipid pattern				
Total cholesterol, mg/dL	**−32 (−62–(−1)), 0.041**	−25 (−56–6), 0.11	−4 (−11–3), 0.22	
Triglycerides, mg/dL	−40 (−109–29), 0.26		−14 (−30–2), 0.086	−11 (−28–4), 0.15
HDL cholesterol, mg/dL	−3 (−15–9), 0.62		0.2 (−3–3), 0.91	
LDL cholesterol, mg/dL	23 (−70–117), 0.62		5 (−16–27), 0.62	
LDL:HDL cholesterol ratio	0.36 (−1–2), 0.61		0.05 (−0.3–0.4), 0.75	
Non-HDL cholesterol, mg/dL	−28 (−59–2), 0.068	−28 (−59–3), 0.080	−4 (−11–3), 0.21	
Lipoprotein (a), mg/dL	52 (−11–115), 0.11	32 (−32–96), 0.32	6 (−8–20), 0.41	
Apolipoprotein A1, mg/dL	−18 (−42–7), 0.15	−8 (−32–17), 0.53	−3 (−9–2), 0.28	
Apolipoprotein B, mg/dL	−8 (−43–26), 0.64		0.8 (−7–9), 0.85	
ApoB:Apo A ratio	0.02 (−0.2–0.2), 0.86		0.01 (−0.03–0.06), 0.57	
Apolipoprotein C-III, mg/dL	4 (−0.2–8), 0.065	4 (−0.7–8), 0.11	**1 (0.4–2), 0.007**	**1 (−0.3–2), 0.009**
Insulin resistance indices *				
Insulin, µU/mL	5 (−3–13), 0.20		−0.5 (−2–1), 0.57	
C-peptide, ng/mL	1 (−0.9–3), 0.29		−0.1 (−0.6–0.3), 0.56	
HOMA2-IR	0.6 (−0.4–2), 0.22		−0.07 (−0.3–0.2), 0.54	
HOMA2-S%	−24 (−92–45), 0.50		**20 (4–36), 0.014**	**20 (4–36), 0.014**
HOMA2-B%-C-peptide	50 (−17–117), 0.14	35 (−32–103), 0.30	2 (−14–17), 0.82	
Carotid ultrasound				
cIMT, microns	19 (−84–122), 0.72		−19 (−42–4), 0.097	−0.8 (−21–19), 0.94
Carotid plaque, n (%)	0.44 (0.09–2.21), 0.32		**0.56 (0.38–0.84), 0.004**	0.73 (0.48–1.12), 0.15

In this analysis, blood scores are the independent variable. Associations with carotid plaque are shown as odds ratios and 95%CI. HOMA2: homeostatic model assessment; LDL: low-density lipoprotein; HDL: high-density lipoprotein. Adjustment was performed for age, diabetes, waist circumference, and smoking (SIRI); sex, diabetes, and obesity (NLR); and sex and smoking (MLR) and age and smoking (PLR). * Analysis of SIRI’s relationship with glucose homeostasis molecules and insulin resistance indices was only performed in non-diabetic patients and if glucose < 110 (n = 338). Significant *p*-values are depicted in bold.

## Data Availability

The data sets used and/or analyzed in the present study are available from the corresponding author upon request.
